# About some exponential inequalities related to the sinc function

**DOI:** 10.1186/s13660-018-1740-9

**Published:** 2018-06-28

**Authors:** Marija Rašajski, Tatjana Lutovac, Branko Malešević

**Affiliations:** School of Electrical Engineering, University of Belgrade, Belgrade, Serbia

**Keywords:** 33B10, 26D05, Exponential inequalities, sinc function

## Abstract

In this paper, we prove some exponential inequalities involving the sinc function. We analyze and prove inequalities with constant exponents and inequalities with certain polynomial exponents. Also, we establish intervals in which these inequalities hold.

## Introduction

Inequalities related to the sinc function, $\operatorname{sinc}x= \frac{\sin x}{x}$ ($x \neq0 $), occur in many fields of mathematics and engineering [1–7] such as Fourier analysis and its applications, information theory, radio transmission, optics, signal processing, sound recording, etc.

The following inequalities are proved in [8]:

1 $$ \cos^{2}{ \frac{x}{2}}\leq\frac{\sin{x}}{x}\leq \cos^{3}{ \frac{x}{3 }}\leq\frac{2+\cos{x}}{3} $$

for every $x\in ( 0,\pi )$. In [9], the authors considered possible refinements of inequality (1) by a real analytic function $\varphi _{a}(x)= ( \frac{\sin x}{x} ) ^{ a} $ for $x\in ( 0,\pi ) $ and parameter $a\in \mathbb{R} $ and proved the following inequalities:

### Statement 1

([9], Theorem 10)

*For all* ${x \in (0, \pi )}$ *and* $a \in (1, \frac{3}{2} )$,

2 $$ \cos^{2}{\frac{x}{2}} \leq \biggl( \frac{\sin{x}}{x} \biggr)^{a} \leq \frac{\sin{x}}{x}. $$

In [9], based on the analysis of the sign of the analytic function

$$F_{a}(x) = \biggl( \frac{\sin x}{x} \biggr) ^{a}- \cos^{2}{\frac{x}{2}} $$

in the right neighborhood of zero, the corresponding inequalities for parameter values $a \geq \frac{3}{2} $ are discussed.

In this paper, in Sect. 3.1, using the power series expansions and the Wu–Debnath theorem, we prove that inequality (2) holds for $a = \frac{3}{2} $. At the same time, this proof represents another proof of Statement 1. Also, we analyze the cases $a\in ( \frac{3}{2} ,2 )$ and $a \geq2$ and prove the corresponding inequalities.

In Sect. 3.2, we introduce and prove a new double-sided inequality of similar type involving polynomial exponents.

Finally, in Sect. 3.3, we establish a relation between the cases of constant and polynomial exponents.

## Preliminaries

In this section, we review some results that we use in our study.

In accordance with [10], the following expansions hold:

3 $$\begin{aligned}& \ln\frac{\sin x}{x} = -\sum_{k=1}^{\infty}{ \frac{2^{2k-1} \vert \mathbf{B}_{2k} \vert }{k(2k)!}x^{2k}} \quad(0 < x < \pi), \end{aligned}$$

4 $$\begin{aligned}& \ln\cos x = -\sum_{k=1}^{\infty}{ \frac{2^{2k-1}(2^{2k}-1) \vert \mathbf {B}_{2k} \vert }{k(2k)!}x^{2k}} \quad(-\pi/2 < x < \pi/2), \end{aligned}$$

where $\mathbf{B}_{i}$ ($i \in {\mathbb {N}}$) are Bernoulli’s numbers.

In our proofs, we use the following theorem proved by Wu and Debnath [11].

### Theorem WD

([11], Theorem 2)

*Suppose that* $f(x)$ *is a real function on* $(a,b)$ *and that* *n* *is a positive integer such that* $f ^{(k)}(a+)$, $f^{(k)}(b-)$ ($k \in\{0,1,2, \ldots,n\}$) *exist*.

(i) *Suppose that* $(-1)^{(n)} f^{(n)}(x)$ *is increasing on* $(a,b)$. *Then*, *for all* $x \in(a,b)$, *we have the following inequality*:
  5 $$ \begin{aligned}[b] &\sum_{k=0}^{n-1} \frac{f^{(k)}(b-)}{k!} (x-b)^{k} + \frac{1}{(a-b)^{n}} \Biggl( f(a + ) - \sum_{k=0}^{n-1} \frac{(a-b)^{k}f^{(k)}(b-)}{k!}\Biggr) (x-b)^{n} \\ &\quad< f(x) < \sum_{k=0}^{n} \frac{f^{(k)}(b-)}{k!}(x-b)^{k}. \end{aligned} $$
  *Furthermore*, *if* $(-1)^{n} f^{(n)}(x)$ *is decreasing on* $(a,b)$, *then the reversed inequality of* (5) *holds*.
(ii) *Suppose that* $f^{(n)}(x)$ *is increasing on* $(a,b)$. *Then*, *for all* $x \in(a,b)$, *we have the following inequality*:
  6 $$ \begin{aligned}[b] &\sum_{k=0}^{n} \frac{f^{(k)}(a+)}{k!}(x-a)^{k} \\ &\quad < f(x) \\ &\quad < \sum_{k=0}^{n-1}\frac{f^{(k)}(a+)}{k!} (x-a)^{k} + \frac{1}{(b-a)^{n}} \Biggl( f(b-) - \sum _{k=0}^{n-1} \frac{(b-a)^{k}f^{(k)}(a+)}{k!} \Biggr) (x-a)^{n}. \end{aligned} $$

*Furthermore*, *if* $f^{(n)}(x)$ *is decreasing on* $(a,b)$, *then the reversed inequality of* (6) *holds*.

### Remark 1

Note that inequalities (5) and (6) hold for $n \in {\mathbb {N}}$ and for $n=0$.

Here, and throughout this paper, a sum where the upper bound of summation is lower than its lower bound is understood to be zero.

The following theorem, which is a consequence of Theorem WD, was proved in [12].

### Theorem 2

([12], Theorem 1)

*Let a function* $f:(a,b) \longrightarrow {\mathbb {R}}$ *have the following power series expansion*:

7 $$ f(x) = \sum_{k=0}^{\infty}{c_{k}(x-a)^{k}} $$

*for* $x \in(a,b)$, *where the sequence of coefficients* $\{c_{k}\}_{k \in {\mathbb {N}}_{0}}$ *has a finite number of nonpositive terms*, *and their indices are in the set* $J = \{j_{0},\ldots,j_{\ell}\}$.

*Then*, *for the function*

8 $$ F(x) = f(x)-\sum_{i=0}^{\ell}{c_{j_{i}}(x-a)^{j_{i}}} = \sum_{k \in {\mathbb {N}}_{0} \backslash J}{c_{k}(x-a)^{k}} $$

*and the sequence* $\{C_{k}\}_{k \in {\mathbb {N}}_{0}}$ *of the nonnegative coefficients defined by*

9 $$ C_{k} = \textstyle\begin{cases} c_{k}, & c_{k} > 0, \\ 0, & c_{k} \leq0, \end{cases} $$

*we have*

10 $$ F(x) = \sum_{k=0}^{\infty}{C_{k}(x-a)^{k}} $$

*for every* $x \in(a,b)$.

*Also*, $F^{(k)}(a+)= k! C_{k}$, *and the following inequalities hold*:

11 $$ \begin{aligned}[b] &\sum_{k=0}^{n}{C_{k}(x-a)^{k}}\\ &\quad < F(x) < \sum_{k=0}^{n-1}{C_{k}(x-a)^{k}} + \frac{1}{(b-a)^{n}} \Biggl( F(b-) - \sum_{k=0}^{n-1}{C_{k}(b-a)^{k}} \Biggr) (x-a)^{n} \end{aligned} $$

*for all* $x \in(a, b)$ *and* $n \in {\mathbb {N}}_{0}$, *that is*,

12 $$ \begin{aligned}[b] &\sum_{k=0}^{m}{ C_{k}} {(x-a)^{k}} + \sum_{i=0}^{\ell}{ c_{j_{i}}} {(x - a)^{j_{i}}} \\ &\quad < f(x) \\ &\quad < \sum_{k = 0}^{m - 1}{ C_{k}} {(x - a)^{k}} + \sum_{i = 0}^{\ell}{ c_{j_{i}}} {(x - a)^{j_{i}}} \\ &\qquad {}+ \frac{{(x - a)}^{m}}{{(b - a)}^{m}} \Biggl( {f(b-) - \sum_{k=0}^{m-1}{ C_{k}} {{(b-a)}^{k}} - \sum_{i=0}^{\ell}{ c_{j_{i}}} {(b-a)}^{j_{i}}} \Biggr) \end{aligned} $$

*for all* $x \in(a,b)$ *and* $m > \max\{j_{0},\ldots,j_{\ell}\}$.

## Main results

### Inequalities with constants in the exponents

First, we consider a connection between the number of zeros of a real analytic function and some properties of its derivatives. It is well known that the zeros of a nonconstant analytic function are isolated [13]; see also [14] and [15].

We prove the following statement.

#### Theorem 3

*Let* $f : (0, c) \longrightarrow {\mathbb {R}}$ *be a real analytic function such that* $f^{(k)}(x) > 0$ *for* $x \in(0,c)$ *and* $k= m, m+1, \ldots$ (*for some* $m \in {\mathbb {N}}$).

*Suppose that the following conditions hold*:

1. *there is a right neighborhood of zero in which* $f(x)<0$, $f'(x)<0$, … , $f^{(m-1)}(x)<0$, *and*
2. $f(c_{-})>0$, $f'(c_{-})>0$, … , $f^{(m-1)}(c_{-})>0$.

*Then there exists exactly one zero* $x_{0} \in(0, c) $ *of the function* *f*.

#### Proof

As $f^{(m)}(x) > 0$ for $x \in(0,c)$, it follows that $f^{(m-1)}(x)$ is an increasing function for $x \in(0,c)$. From conditions (1) and (2) we conclude that there exists exactly one zero $x_{m-1} \in(0, c)$ of the function $f^{(m-1)}(x)$. Next, we can conclude that function $f^{(m-2)}(x)$ is decreasing for $x \in(0,x_{m-1})$ and increasing for $x \in(x_{m-1},c)$. It is clear that the function $f^{(m-2)}(x) $ has exactly one minimum in the interval $(0, c)$ at point $x_{m-1}$ and $f^{(m-2)}(x_{m-1}) < 0$. From condition (2) it follows that the function $f^{(m-2)}(x)$ has exactly one root $x_{m-2}$ on the interval $(0, c)$ and $x_{m-2} \in(x_{m-1}, c)$.

By repeating the described procedure, we get the statement of the theorem. □

Let us consider the family of functions

13 $$ f_{a}(x) = a \ln\frac{\sin x}{x} - 2 \ln\cos\frac{x}{2} $$

for $x \in(0, \pi)$ and parameter $a \in(1, +\infty)$.

Obviously, the following equivalence is true:

14 $$ a_{1} < a\quad \Longleftrightarrow \quad f_{a}(x) < f_{a_{1}}(x) $$

for $a, a_{1} > 1 $ and $x \in(0,\pi)$.

Thus

15 $$ \frac{3}{2} < a\quad \Longleftrightarrow \quad f_{a}(x) < f_{\frac{3}{2}}(x) \quad\mbox{for }x \in(0,\pi). $$

By the power series expansions (3) and (4), we have

16 $$ f_{a}(x) = \sum_{k=1}^{\infty}{E_{k} x^{2k}} $$

for $a>1$ and $x \in(0, \pi)$, where

17 $$ E_{k} = \frac{((2-a) 4^{k}-2 ) \vert \mathbf{B}_{2k} \vert }{2 k \cdot(2k)!} \quad(k \in {\mathbb {N}}). $$

For $a = \frac{3}{2} $, we have $E_{1}=0$ and $E_{k}>0$ for $k=2,3 , \ldots$ . Thus from (16) we have

$$f_{\frac{3}{2}}(x)> 0 \quad \mbox{for } x \in(0, \pi), $$

and consequently we have the following result.

#### Theorem 4

*For all* $x \in(0, \pi)$, *we have*

$$\cos^{2}{ \frac{x}{2}} \leq \biggl(\frac{\sin{x}}{x} \biggr)^{\frac{3}{2}} \leq \frac{\sin{x}}{x}. $$

Since

$$\biggl(\frac{\sin{x}}{x} \biggr)^{\frac{3}{2}} \leq \biggl(\frac {\sin{x}}{x} \biggr)^{a} $$

for $x \in(0, \pi)$ and $a \in (1, \frac{3}{2} ]$, the previous theorem can be thought of as a new proof of Statement 1.

Consider now the family of functions $f_{a}(x) = a \ln\frac{\sin x}{x} - 2 \ln\cos\frac{x}{2} $ for $x \in(0, \pi)$ and parameter $a > \frac{3}{2} $.

It easy to check that for the sequence

18 $$ \alpha_{k}=2-\frac{2}{4^{k}},\quad k \in {\mathbb {N}}, $$

the following equivalences are true:

19 $$ \begin{aligned}[b] &a = \alpha_{k}\quad \Longleftrightarrow \quad E_{k} = 0, \\ &a \in (\alpha_{k}, \alpha_{k+1} )\quad \Longleftrightarrow \quad \bigl( \forall i \in\{1,2,\ldots,k \} \bigr)\ E_{i} < 0 \wedge (\forall i > k )\ E_{i} > 0. \end{aligned} $$

Let us now consider the function $\mathfrak{m} : [ \frac{3}{2} ,2 ) \longrightarrow {\mathbb {N}}_{0}$ defined by

20 $$ \mathfrak{m}(a)=k \quad\mbox{if and only if}\quad a \in ( \alpha_{k}, \alpha_{k+1} ]. $$

It is not difficult to check that $\lim_{a \rightarrow2_{-}} \mathfrak{m}(a) = +\infty$, whereas for a fixed $a \in (\frac{3}{2} ,2 )$, the number of negative elements of the sequence $\{ E_{k} \}_{k \in {\mathbb {N}}}$ is $\mathfrak{m}(a)$, and their indices are in the set $\{ 1, \ldots, \mathfrak{m}(a)\}$. For this reason, we distinguish two cases $a \in ( \frac{3}{2} ,2 )$ and $a\geq2$.

As for the parameter $a = 2$ and $x \in(0, \pi)$, we have

$$\biggl(\frac{\sin{x}}{x} \biggr)^{ 2} \leq\cos^{2}{ \frac{x}{2}}\quad \Longleftrightarrow \quad \sin^{2}\frac{x}{2} \leq \biggl(\frac {x}{2} \biggr)^{2}, $$

whereas for $a>2$ and $x \in(0, \pi)$, we have

$$\biggl(\frac{\sin{x}}{x} \biggr)^{ a} \leq \biggl(\frac{\sin {x}}{x} \biggr)^{ 2}. $$

Hence, we have proved the following theorem.

#### Theorem 5

*For all* $a \geq2$ *and* $x \in (0, \pi)$, *we have*

21 $$ \biggl(\frac{\sin{x}}{x} \biggr)^{ a} \leq \cos^{2}{ \frac{x}{2}}. $$

Consider now the case where the parameter $a \in ( \frac{3}{2} ,2 ) $. As noted before, for any fixed $a \in ( \frac{3}{2} ,2 ) $, there is a finite number of negative coefficients in the power series expansion (17), so it is possible to apply Theorem 2.

According to Theorem 2, we have the following inequalities:

22 $$ \begin{aligned}[b] &\sum_{k=\mathfrak{m}(a)+1}^{n} {E_{k}} {x^{k}} + \sum_{i=0}^{\mathfrak{m}(a)-1}{ E_{i}} {x^{i}} \\ &\quad< f_{a}(x) \\ &\quad< \Biggl({f_{a}(c-) - \sum_{k=\mathfrak{m}(a)+1}^{n-1}{E_{k}} {c^{k}} - \sum_{i=0}^{\mathfrak{m}(a)-1}{E_{i}} {c^{i}}} \Biggr) \frac{{x}^{n}}{{c}^{n}} + \sum _{k=\mathfrak{m}(a)+1}^{n-1}{E_{k}} {x^{k}} + \sum _{i=0}^{\mathfrak{m}(a)-1}{ E_{i}} {x^{i}} \end{aligned} $$

for all $x \in (0,c )$, $c \in (0,\pi )$, $n > \mathfrak{m}(a)+1$, and $a \in ( \frac{3}{2} ,2 )$.

The family of functions $f_{a}(x)$ for $x \in(0, \pi)$ and $a \in ( \frac{3}{2} ,2 )$ satisfy conditions (1) and (2) of Theorem 3, as we prove in the following lemma.

#### Lemma 1

*Consider the family of functions* $f_{a}(x) = a \ln\frac{\sin x}{x} - 2 \ln\cos\frac{x}{2} $ *for* $x \in(0,\pi)$ *and parameter* $a \in ( \frac{3}{2} ,2 )$. *Let* $m=\mathfrak{m}(a)$, *where* $\mathfrak{m}(a)$ *is defined as in* (20).

*Then* $\frac{d^{k}}{dx^{k}} f_{a}(x) > 0$ *for* $k = m, m+1, \ldots $ *and* $x \in(0,\pi)$, *and the following assertions hold*:

1. *There is a right neighborhood of zero in which the following inequalities hold*: $f_{a}(x)<0$, $\frac{d}{dx} f_{a}(x)<0$, … , $\frac{d^{m-1}}{dx^{m-1}} f_{a}(x)<0$;
2. $f_{a}(\pi_{-})>0$, $\frac{d}{dx}f_{a}(\pi_{-})>0$, … , $\frac{d^{m-1}}{dx^{m-1}} f_{a}(\pi_{-})>0$.

#### Proof

Let us recall that, for any fixed $a \in ( \frac{3}{2} ,2 ) $, there is a finite number of negative coefficients in the power series expansion (17). Also, we have

$$\biggl( \frac{d}{dx} f_{a} \biggr) (x) = a \biggl( \cot x - \frac{1}{x} \biggr) + \tan\frac{x}{2}. $$

For the derivatives of the function $f_{a}(x)$ in the left neighborhood of *π*, it suffices to observe that

$$\biggl( \frac{d}{dx} f_{a} \biggr) (\pi-x) = a \biggl( -\cot x - \frac{1}{\pi-x} \biggr) + \cot\frac{x}{2} = \frac{2-a}{x}- \frac{a}{\pi}+ \biggl( a \biggl( \frac{1}{3}-\frac {1}{\pi^{2}} \biggr)-\frac{1}{6} \biggr) x+\cdots . $$

From this the conclusions of the lemma can be directly derived. □

Thus, for every $a \in ( \frac{3}{2} ,2 )$, the corresponding function $f_{a}(x) = a \ln\frac{\sin x}{x} - 2 \ln\cos\frac{x}{2} $ has exactly one zero on the interval $(0, \pi)$. Let us denote it by $x_{a}$.

The following theorem is a direct consequence of these considerations.

#### Theorem 6

*For every* $a \in ( \frac{3}{2} ,2 )$ *and all* $x \in (0, x_{a} ]$, *where* $0< x_{a} < \pi$, *we have*

23 $$ \biggl(\frac{\sin{x}}{x} \biggr)^{ a} \leq \cos^{2}{ \frac{x}{2}}. $$

For the selected discrete values of $a \in ( \frac{3}{2} , 2 ) $, the zeros $x_{a}$ of the corresponding functions $f_{a} (x)$ are shown in Table 1. Although the values $x_{a}$ can be obtained by any numerical method, the following remark can also be used to locate them.

**Table 1:** Values $x_{a}$ and $m_{a}$ for some specified $a \in ( \frac{3}{2} ,2 ) $ related to Theorems 6 and 10, respectively

*a*	1.501	1.502	1.503	1.504	1.505	1.506	1.507	1.508	1.509	1.510
$x_{a}$	0.282…	0.398…	0.487…	0.561…	0.626…	0.685…	0.738…	0.788…	0.834…	0.878…
$m_{a}$	0.140…	0.198…	0.243…	0.280…	0.314…	0.344…	0.371…	0.397…	0.421…	0.444…
*a*	1.52	1.53	1.54	1.55	1.56	1.57	1.58	1.59	1.60	1.65
$x_{a}$	1.220…	1.468…	1.666…	1.831…	1.973…	2.096…	2.205…	2.302…	2.302…	2.302…
$m_{a}$	0.628…	0.769…	0.888…	0.993…	1.088…	1.175…	1.256…	1.256…	1.256…	1.256…
*a*	1.70	1.75	1.80	1.85	1.90	1.92	1.94	1.96	1.98	1.9999
$x_{a}$	2.911…	3.034…	3.103…	3.133…	3.141…	3.141…	3.141…	3.141…	3.141…	3.141…
$m_{a}$	1.986…	2.221…	2.433…	2.628…	2.809…	2.879…	2.947…	3.013…	3.087…	3.141…

#### Remark 7

For a fixed $a \in ( \frac{3}{2} ,2 )$, select $n>\mathfrak{m}(a)+1$ and consider inequalities (22). Denote the corresponding polynomials on the left- and right-hand sides of (22) by $P_{L}(x)$ and $P_{R}(x)$, respectively. These polynomials are of negative sign in a right neighborhood of zero (see [15], Theorem 2.5), and they have positive leading coefficients. Then, the root $x_{a}$ of the equation $f_{a}(x) = 0$ is always localized between the smallest positive roots of the equations $P_{L} (x) = 0$ and $P_{R} (x) = 0$.

### Inequalities with polynomial exponents

In this subsection, we propose and prove a new double-sided inequality involving the sinc function with polynomial exponents.

To be more specific, we find two polynomials of the second degree that, when placed in the exponent of the sinc function, give an upper and a lower bound for ${\cos}^{2} \frac{x}{2}$.

#### Theorem 8

*For every* $x\in(0,3.1)$, *we have the double*-*sided inequality*

24 $$ { \biggl( {\frac{{\sin x}}{x}} \biggr)^{ {p_{1}} ( x )}} < { \cos}^{2} \frac{x}{2} < { \biggl( {\frac{{\sin x}}{x}} \biggr)^{ {p_{2}} ( x )}}, $$

*where* ${p_{1}} ( x ) = \frac{3}{2} + \frac{{{x^{2}}}}{{2{\pi^{2}}}} $ *and* ${p_{2}} ( x ) = \frac{3}{2} + \frac{{{x^{2}}}}{{80}} $.

#### Proof

Consider the equivalent form of inequality (24)

$${p_{1}} ( x )\ln\frac{{\sin x}}{x} < 2\ln\cos\frac{x}{2} < {p_{2}} ( x )\ln\frac{{\sin x}}{x}. $$

Now, let us denote

$${G_{i}} ( x ) = {p_{i}} ( x )\ln\frac{{\sin x}}{x} - 2\ln \cos\frac{x}{2} $$

for $i=1,2$.

Based on Theorem WD, from (3) we obtain

25 $$ \begin{aligned}[b] &-\sum _{k=1}^{m-1}{ \frac{2^{2k-1} \vert B_{2k} \vert }{k(2k)!} x^{2k}} + \biggl( \frac{1}{c} \biggr)^{ 2m} \Biggl( \ln \frac{\sin c}{c} - \sum_{k=1}^{m-1}{ \frac{2^{2k-1} \vert B_{2k} \vert }{k(2k)!} c^{2k}} \Biggr) x^{2m} \\ &\quad< \ln \frac{\sin x}{x} < -\sum_{k=1}^{n}{ \frac{2^{2k-1} \vert B_{2k} \vert }{k(2k)!} x^{2k}} \end{aligned} $$

for $x \in (0,\pi )$, where $n, m \in {\mathbb {N}}$, $m,n \ge2$.

Based on Theorem WD, from (4) we obtain

26 $$ \begin{aligned}[b] &- \sum_{k=1}^{m-1}{ \frac{2^{2k-1}(2^{2k}-1) \vert B_{2k} \vert }{k(2k)!} x^{2k}} + \biggl( \frac{1}{c} \biggr)^{ 2m} \Biggl( \ln\cos c - \sum_{k=1}^{m-1}{ \frac{2^{2k-1}(2^{2k}-1) \vert B_{2k} \vert }{k(2k)!} c^{2k}} \Biggr) x^{2m} \\ &\quad < \ln\cos x < - \sum_{k=1}^{n}{ \frac{2^{2k-1}(2^{2k}-1) \vert B_{2k} \vert }{k(2k)!} x^{2k}} \end{aligned} $$

for $x \in (0,c )$, where $0 < c < \frac{\pi}{2} $, $n, m \in {\mathbb {N}}$, $m,n \ge2$, that is,

27 $$ \begin{aligned}[b] & \sum _{k=1}^{n}{ \frac{(2^{2k}-1) \vert B_{2k} \vert }{2k(2k)!} x^{2k}} \\ &\quad < - \ln \cos\frac{x}{2} \\ &\quad < \sum_{k=1}^{m-1}{ \frac{(2^{2k}-1) \vert B_{2k} \vert }{2k(2k)!} x^{2k}} - \biggl( \frac{2}{c} \biggr)^{ 2m} \Biggl( \ln \cos\frac{c}{2} - \sum_{k=1}^{m-1}{ \frac{(2^{2k}-1) \vert B_{2k} \vert }{2k(2k)!} c^{2k}} \Biggr) x^{2m} \end{aligned} $$

for $x \in (0,c )$ and $0 < c < \pi$, $n, m \in {\mathbb {N}}$, $m,n \ge2$.

Now, let us introduce the notation

$$\begin{aligned}[b] &{H_{1}} ( {x,{m_{1}},{n_{1}},c_{1}} ) \\ &\quad = - {p_{1}} ( x ) \sum_{k = 1}^{{m_{1}} - 1} \frac{{{2^{2k - 1}} \vert {{B_{2k}}} \vert }}{{k(2k)!}}{x^{2k}} \\ &\qquad{}- 2 \Biggl( { - \sum_{k = 1}^{{m_{1}} - 1} \frac{{ ( {{2^{2k}} - 1} ) \vert {{B_{2k}}} \vert }}{{2k(2k)!}}{x^{2k}} + \frac{1}{{{c_{1}^{2m_{1}}}}} \Biggl( {\ln \frac {c_{1}}{2} + \sum_{k = 1}^{{n_{1}} - 1} \frac{{ ( {{2^{2k}} - 1} ) \vert {{B_{2k}}} \vert }}{{2k(2k)!}}{c_{1}^{2k}}} \Biggr){x^{2m_{1}}}} \Biggr) \end{aligned} $$

for $m_{1},n_{1}\in {\mathbb {N}}$, $m_{1},n_{1} \ge2$, $c_{1}\in(0,\pi)$, and $x \in(0,c_{1})$;

$$\begin{aligned}[b] &{H_{2}} ( {x,{m_{2}},{n_{2}},c_{2}} )\\ &\quad = {p_{2}} ( x ) \Biggl( { - \sum_{k = 1}^{{m_{2}} - 1} \frac{{{2^{2k - 1}} \vert {{B_{2k}}} \vert }}{{k(2k)!}}{x^{2k}} + \frac {1}{{{c_{2}^{2m_{2}}}}} \Biggl( {\ln \frac{{\sin c_{2}}}{c_{2}} + \sum_{k = 1}^{{m_{2}} - 1} \frac{{{2^{2k - 1}} \vert {{B_{2k}}} \vert }}{{k(2k)!}}{c_{2}^{2k}}} \Biggr){x^{2m_{2}}}} \Biggr) \\ &\qquad{}+ 2 \sum_{k = 1}^{{n_{2}}} \frac{{ ( {{2^{2k}} - 1} ) \vert {{B_{2k}}} \vert }}{{2k(2k)!}}{x^{2k}} \end{aligned} $$

for $m_{2},n_{2}\in {\mathbb {N}}$, $m_{2},n_{2} \ge2$, $c_{2}\in(0,\pi)$, and $x \in (0,c_{2})$.

By inequalities (25) and (27) we have

$$\begin{gathered} {G_{1}} ( x ) < {H_{1}} ( x, m_{1}, n_{1}, c_{1} ), \\ {G_{2}} ( x ) > {H_{2}} ( x, m_{2}, n_{2}, c_{2} ), \end{gathered} $$

for $m_{1},n_{1},m_{2},n_{2} \in {\mathbb {N}}$ and $c_{1},c_{2} \in(0,\pi)$.

For $c_{1}=c_{2}=3.1$, $m_{1}=25$, and $n_{1}=10$ and for $m_{2}=13$ and $n_{2}=27$, it is easy to prove that ${H_{1}} ( x, m_{1}, n_{1}, c_{1} )<0$ and ${H_{2}} ( x, m_{2}, n_{2}, c_{2} )>0$ for every $x \in(0,c_{1})$.

Hence we conclude that $G_{1}(x)<0$ and $G_{2}(x)>0$ for every $x \in (0,3.1)$, and the double-sided inequality (24) holds. □

#### Remark 9

Note that this method can be used to prove that inequality (24) of Theorem 8 holds on any interval $(0,c)$ where $c \in(0, \pi)$, but the degrees of the polynomials $H_{1}$ and $H_{2}$ get larger as *c* approaches *π*.

### Constant vs. polynomial exponents

Let us observe the inequalities in Theorems 6 and 8 and inequality (24) containing constants and polynomials in the exponents, respectively.

A question of establishing a relation between these functions, with different types of exponents, comes up naturally. The following theorem addresses this question.

#### Theorem 10

*For all* $a \in ( \frac{3}{2} ,2 )$ *and* $x\in (0,m_{a} )$, *where* $m_{a}=\sqrt{2\pi^{2} ( {a - \frac{3}{2}} )}$, *we have the following double*-*sided inequality*:

28 $$ { \biggl( {\frac{{\sin x}}{x}} \biggr)^{ a}} < { \biggl( {\frac{{\sin x}}{x}} \biggr)^{ {\frac{3}{2} + \frac {{{x^{2}}}}{{2{\pi^{2}}}}}} } < {\cos^{2}} \frac{x}{2}. $$

#### Proof

Let $a = \frac{3}{2} + \varepsilon$, $\varepsilon\in (0, \frac{1}{2} )$, and $x>0$. Then

$$\begin{aligned}[b] &\biggl( {\frac{3}{2} + \frac{{{x^{2}}}}{{2{\pi^{2}}}}} \biggr)\ln\frac {{\sin x}}{x} - a\ln\frac{{\sin x}}{x} \\ &\quad = \biggl( { \frac{3}{2} + \frac{{{x^{2}}}}{{2{\pi^{2}}}}} \biggr)\ln \frac{{\sin x}}{x} - \biggl( { \frac{3}{2} + \varepsilon} \biggr)\ln \frac{{\sin x}}{x} \\ &\quad = \biggl( {\frac{{{x^{2}}}}{{2{\pi^{2}}}} - \varepsilon} \biggr)\ln \frac{{\sin x}}{x} = \frac{1}{{2{\pi^{2}}}} \bigl( {x - \sqrt{2{\pi ^{2}}\varepsilon}} \bigr) \bigl( {x + \sqrt{2{\pi^{2}}\varepsilon}} \bigr)\ln\frac{{\sin x}}{x}. \end{aligned} $$

Now we have

$$\begin{aligned} x \in \bigl( {0,\sqrt{2{\pi^{2}}\varepsilon}} \bigr) &\quad \Longleftrightarrow\quad \biggl({ \frac{3}{2} + \alpha{x^{2}}} \biggr) \ln\frac{{\sin x}}{x} > \biggl( { \frac{3}{2} + \varepsilon} \biggr)\ln \frac{{\sin x}}{x}\\ &\quad \Longleftrightarrow \quad { \biggl( {\frac{{\sin x}}{x}} \biggr)^{ \frac{3}{2} + \frac {{{x^{2}}}}{{2{\pi^{2}}}}}} > { \biggl( {\frac{{\sin x}}{x}} \biggr)^{ \frac{3}{2} + \varepsilon}}. \end{aligned} $$

Hence, applying Theorem 8, the double-sided inequality (28) holds for all ${a \in ( \frac{3}{2} ,2 )}$ and $x \in(0,m_{a})$. □

In Table 1 we show the values $x_{a}$ and $m_{a}$ for some specified $a \in ( \frac{3}{2} ,2 ) $.

#### Remark 11

Note that Theorem 10 represents another proof of the following assertion from [9]:

$$\bigl( \forall a \in (3/2,2 ) \bigr)\ ( \exists\delta> 0 )\ \bigl( \forall x \in(0, \delta)\bigr)\quad { \biggl( {\frac{{\sin x}}{x}} \biggr)^{ a}} < { \cos^{2}}\frac{x}{2}. $$

## Conclusion

In this paper, using the power series expansions and the application of the Wu–Debnath theorem, we proved that inequality (2) holds for $a = \frac{3}{2} $. At the same time, this proof represents a new short proof of Statement 1.

We analyzed the cases $a \in ( \frac{3}{2} ,2 )$ and $a \geq 2$, and we proved the corresponding inequalities. We introduced and proved a new double-sided inequality of similar type involving polynomial exponents. Also, we established a relation between the cases of constant and polynomial exponents.
